# George Marinesco in the Constellation of Modern Neuroscience

**DOI:** 10.3389/fnins.2017.00726

**Published:** 2017-12-25

**Authors:** Ioan Opris, Valeriu S. Nestianu, Adrian Nestianu, Liviu Bilteanu, Jean Ciurea

**Affiliations:** ^1^Miller School of Medicine, University of Miami, Miami, FL, United States; ^2^University of Medicine and Pharmacy of Craiova, Craiova, Romania; ^3^University of Medicine and Pharmacy Carol Davila, Bucharest, Romania; ^4^Bagdasar Arseni Hospital, Bucharest, Romania

**Keywords:** Marinesco bodies, Marinesco-Radovici reflex, Marinesco-Sjögren syndrome, Alzheimer disease, Parkinson's disease, senil plaques, substantia nigra, nervous cell

## Abstract

George Marinesco is the founder of Romanian School of Neurology and one of the most remarkable neuroscientists of the last century. He was the pupil of Jean-Martin Charcot in Salpêtrière Hospital in Paris, France, but visited many other neurological centers where he met the entire constellation of neurologists of his time, including Camillo Golgi and Santiago Ramón y Cajal. The last made the preface of Nervous Cell, written in French by Marinesco. The original title was “La Cellule Nerveuse” and is considered even now a basic reference book for specialists in the field. He was a refined clinical observer with an integrative approach, as could be seen from the multitude of his discoveries. The descriptions of the succulent hand in syringomyelia, senile plaque in old subjects, palmar jaw reflex known as Marinesco-Radovici sign, or the application of cinematography in medicine are some of his important contributions. He was the first who described changes of locus niger in a patient affected by tuberculosis, as a possible cause in Parkinson disease. Before modern genetics, Marinesco and Sjögren described a rare and complex syndrome bearing their names. He was a hardworking man, focused on his scientific research, did not accepted flattering of others and was a great fighter against the injustice of the time.

## Introduction

### Biographical notes

George Marinesco (in Romanian Gheorghe Marinescu) is the founder of Romanian School of Neurology and one of the most remarkable neuroscientists of the twentieth century. He was born on February 28, 1863 in Bucharest and died on May 15, 1938 in Bucharest, Romania (Voiculescu, 1970).

### Early studies

In 1882, after graduating from the Central Seminary, he enrolled at the Faculty of Medicine of “Carol Davila” University of Medicine and Pharmacy in Bucharest. After the graduation of Medical School, Marinesco received most of his medical skills in the laboratory of histology from the Brâncoveanu Hospital and as assistant at the Bacteriological Institute under Victor Babes, who already published on myelitis transversa, hysterical muteness, and pupil dilatation in pneumonia.

### From babes to charcot

Victor Babes understood that Marinesco needs more for his professional ground and offered him the possibility to study abroad. The place is remarkable and visionary chosen: “Salpêtrière” Hospital in Paris headed by Jean Marie Charcot who will leave the most important mark on the young scientist. On Babes recommendation, in 1889 the Romanian government awarded him a grant in Paris to undertake postgraduate training in neurology under Jean-Martin Charcot. Marinesco recognized the powerful influence of his master all his life and paid tribute always when possible to his Initiator and Master in Neuroscience. This consideration was mutually manifested. As a proof of this aspect, Marinesco presented the results of Charcot and coworkers on acromegaly in a scientific meeting in Berlin in 1890. Years later, he was chosen by Charcot's disciples to speak on his commemoration. At the Salpêtrière Hospital he meets Pierre Marie, with whom he will maintain close ties in the future, Joseph Babinski and Fulening Raymond.

After 9 years of successful scientific activity abroad (in Paris and other places), where he met the entire constellation of Neurology from Europe, Marinesco returned to Bucharest, in 1897, to begin a new professorial department at Pantelimon Hospital, created for him (Marinesco, 1897a,b). Shortly after, in the same year, it was created a chair position of Clinical Neurology at the University of Bucharest (now “Carol Davila” University of Medicine and Pharmacy), in the Colentina Hospital. He kept this position for the next 41 years, being regarded as the “founder of the Romanian School of Neurology” (Ionita and Fine Edward, 2003)

### Marinesco's personality

Professor Marinesco had an enigmatic “sphinx”—like face, as seen from pictures and spoken history. He did not speak much, but was able to create a powerful impression of equilibrium since the first contact. Friends and his pupils presented him as a determined person, with a warm soul, high spirit, very good communication, concerned with his scientific opinions, and well-tempered with spontaneous irony. He was gifted with a huge work capacity. He arrived early in the morning at his office and left the Department late in night. There is a story on Marinesco and Sager, telling that Oscar was already on Department when Professor arrived at 7 o'clock in the morning. The next day Professor arrived earlier and Oscar Sager was there. This happened for the next days too till Oscar Sager clearly understood that nobody must come earlier than Marinesco. This became a tradition in Romanian Neurology and Neurosurgery till our days.

Professor Marinesco was so busy, he dined once a day in the same office/laboratory (microscopy room). His regular lunch consisted of two hard boiled eggs and took no more than few minutes. Since he became well known, many newspaper men/journalists wished to visit and interview him. He kindly accepted, shacked hands with them above his imposing wood desk, inviting them to sit, and after only few minutes rose up and very elegantly said: “*Gentlemen, I know you must be busy, I don't wish to take your time…*” Time must be saved for research, no matter how.

Marinesco was extremely dedicated to his intensive, dense work. His humble family origin and his Seminary education gave him a tool to understand those in need and suffer. Science and empathy were both present in him. All those characteristics revealed not only the scientist, but also a pleasant person as perceived by his contemporaries.

## George marinesco is the first romanian neuroscientist

Marinesco was aware of Sherrington's hypothesis on cell membrane polarization and recognized this possibility in his book “*The Nervous Cell*” (Marinesco, 1909). Meanwhile, in the same book, he mentions the axonal tubules as a route for signal path. Keeping a certain reserve, it is still worth to mention “Orch-Or” hypothesis referring to the same structure as a quota signal path support.

The electromagnetic phenomena of living matter are captivating human mind ever since antiquity. Only later this activity was reproduced, measured, and analyzed. The founder of Romanian Experimental Neurophysiology was Professor Ioan Athanasiu from Veterinary Medical School in Bucharest, a friend of Marinesco, who was a pupil and co-worker of Marey, and also of Willem Einthoven. It was the early beginning of twentieth century when striate and cardiac muscle activity were studied in Bucharest by Athanasiu and Marinesco using an early version of electrocardiograph and electromyograph. They understood the normal activity and later extended to patients with myastenia, paresis and other motor deficits. The team of Athanasiu and Marinesco will be soon larger, including Oscar Sager (the author of the monography “Thalamus”) and Arthur Kraindler.

Since this galvanometer with string was mechanically very sensitive and influenced even by cars passing on nearby roads, the group of enthusiastic scientists decided to work late on the night. That is why their research was seen by outsiders as “mysterious.” It was, in fact, a mystery, the scientists will clarify this later. Marinesco tried to record brain activity with the same galvanometer before Hans Berger (see Haas, 2003), but he did not accomplish this due to the difference in signal amplitude. Then, he bought an electroencephalograph (EEG) with light printer on photopaper (having just one channel). He and his team, recorded healthy subjects for training and for understanding normal activity and then they focused on patients. A detailed description of alfa rhythm, spindle activity occurrence condition of appearance and disappearance (such as eyes opening, or intermittent light stimulation) could be considered as Romanian contributions to this field “*en première*.” Slow EEG waves produced by brain mass lesions, epileptic seizure activity, but mainly comatose encephalitic states of brain bioelectric activities were studied and described. All the progress was performed mainly in “Colentina” Hospital, where Marinesco was the Head of Neurological Department.

He was a member of the Romanian Academy in his mid-thirties, when he succeeded to transmit brain's bioelectrical activity from a patient monitored in his Department through a phone line to a meeting in Romanian Academy Aula, an accomplishment ahead of its time. The Polytechnic section participants smiled when they said “Dr. Marinesco proved once again that currents are passing through wires.” Despite of this “cruel” comment, it is obvious that he was a clear fore-viewer in the field of telemedicine with neuroscientific application.

He is extending his bioelectrical studies to behavior and emotion in cooperation with Louis Copelman. Moreover, his area of interest is extended to parapsychology, metaphysics, and esoteric zone. This was not unexpected for a neuroscientist of that time, when fundamental existential questions prevailed in intellectual groups.

## The constellation of the founders of modern neurology

Marinesco was in the privileged position to study with Jean-Martin Charcot (Marinesco, 1925), the founder of French School of Neurology in Salpêtrière Hospital in Paris, France. Here, he establishes close ties with Pierre Marie, Joseph Babinski, and Fulening Raymond. He collaborated with French pathologists Paul Oscar Blocq and Édouard Brissaud. Later, he learned histology techniques from Carl Weigert in Frankfurt and electrophysiology from Emil du Bois-Reymond in Berlin.

**Figure 1:**
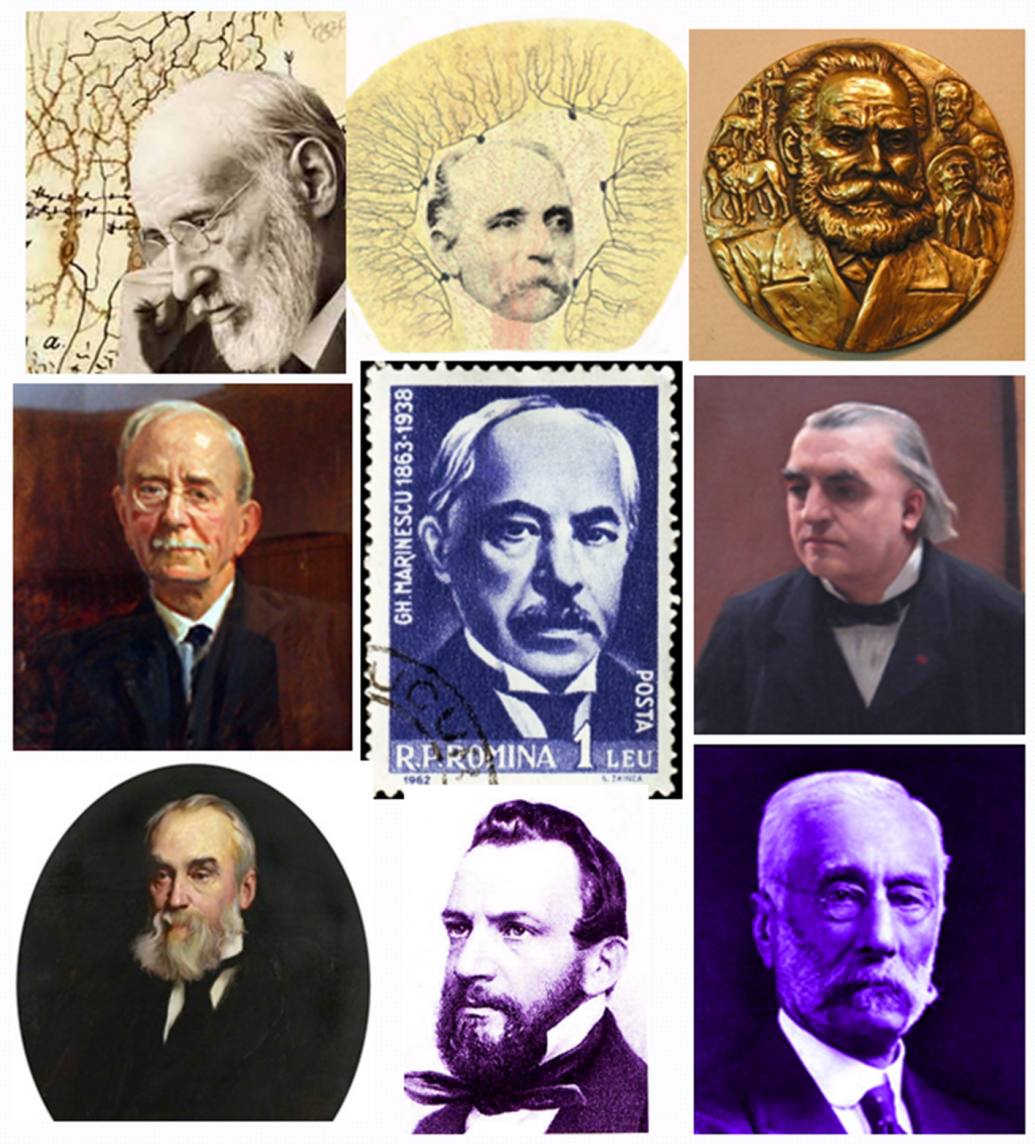
The constellation of the founders of modern neuroscience. The picture depicts George Marinesco surrounded by the greatest neuroscientists of the time. The top row from left to right has Santiago Ramon y Cajal, Camillo Golgi, and Ivan Pavlov, The middle row has Charles Sherrington on the left and Charcot on the right of Marinesco. On the lower row are Hughlings Jackson, Emil du Bois-Reymond) and David Ferrier. Images were taken Wikipedia.

Between 1890 and 1896 he traveled to Spain, Germany, England, Belgium, and Italy. He met Santiago Ramon Y Cajal in Spain, Golgi and Mingazzini in Italy, Bekhterev, Korsakow, and Kojewnikow in Russia, Hughlings Jackson and Sir Victor Horsley in United Kingdom, von Monakow and Forel in Switzerland (Figure 1). His travels were supported by French journal “Medical Week” “La Semaine Médicale” in French, to inform on foreign schools of Neurology (Marinesco et al., 1984). Then, he worked with Carl Weigert in Frankfurt am Main with Emil du Bois-Raymond in Berlin, and also with Hermann Oppenheim and Wilhelm Heinrich Erb.

The greatest scholars in European Neurology were involved in writing the Jubilee Volume published in Marinesco's honor in 1933. Santiago Ramón y Cajal “cordially” congratulated “the famous Romanian investigator” and stated that “his work has been fruitful and in multiple new topics” (Marinesco, 1933). Barre (1933), Professor of Clinical Neurology in Strasbourg, ends his contribution by sending to Marinesco “*the homage of our admiration for the magnificent effort he continues to pursue in all areas of neurology, the high originality of his conceptions and the proven value of the knowledge that we owe*” (Catala and Poirier, 2012).

Marinesco had a huge admiration for Pavlov's work. His book (Marinesco and Kreindler, 1935) was devoted to “conditional reflexes” and dedicated “to the great physiologist I.P. Pavlov” with the following words: “*The doctrine of conditional reflexes, as was inaugurated by Pavlov, Bekhterev and their students, not only casts a bright light on the physiological processes that occur in the cerebral cortex, but psychology, psychiatry and nervous pathology have largely benefited from these discoveries. We believe that this is only the beginning*.”

Marinesco's monography *Le Tonus des Muscles striés* (Marinesco, 1937) written together with Nicolae Ionescu-Siseşti, Oskar Sager, and Arthur Kreindler, was endorsed by one of the most respected scientists, Sir Charles Sherrington, who wrote the preface/foreword.

In the opening remarks of the XVII-th International Neurological Meeting, George Bourguignon (1938), the President of the Society of Neurology of Paris, pays tribute to Marinesco whose death has just been announced. He praises Marinesco's merits both as a scientist and as a great friend of France (Bourguignon, 1938).

### Marinesco's work has been endorsed by santiago ramón y cajal

**Figure 2:**
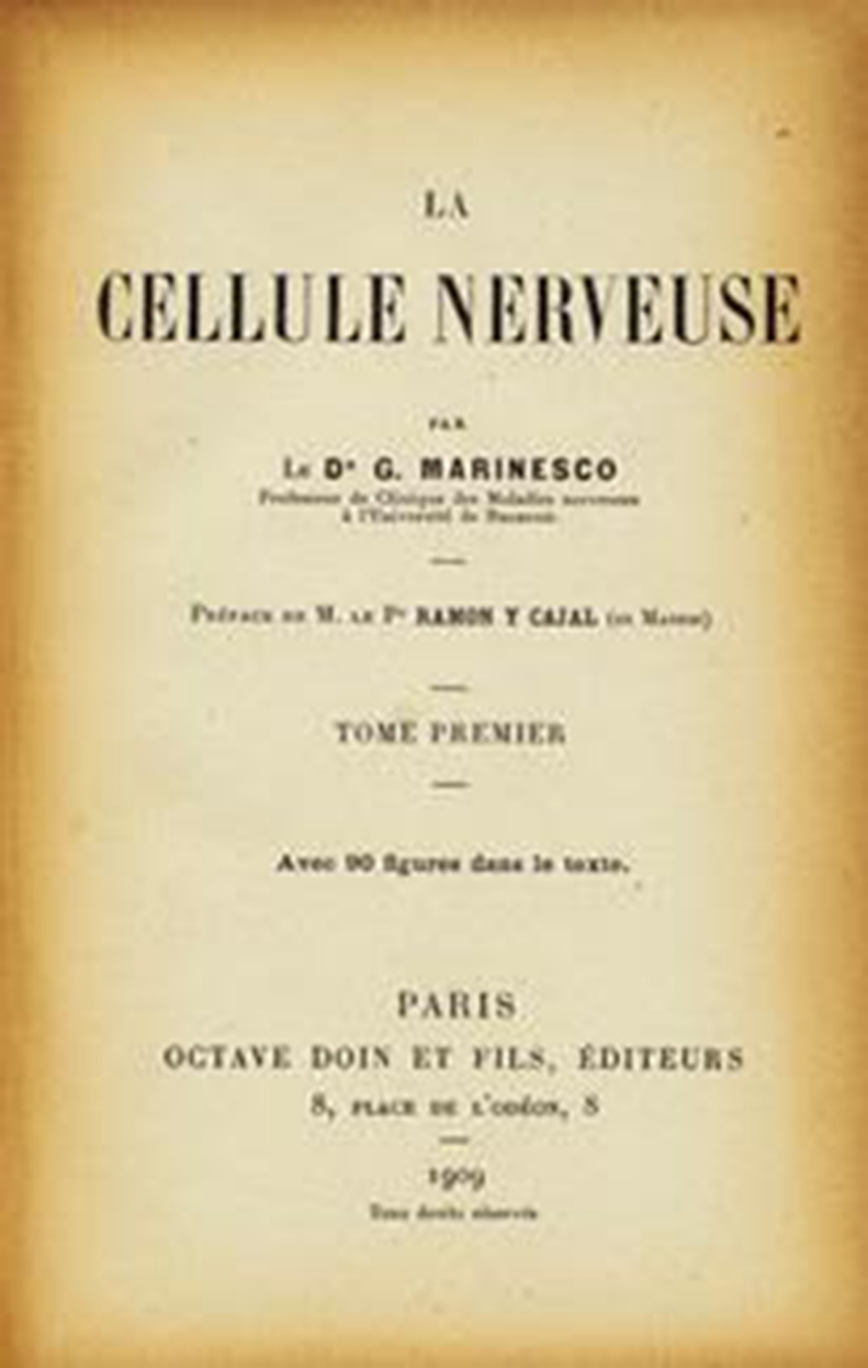
Flyleaf of the book “La Cellule Nerveuse” published by Marinesco (1909).

In 1909 the monumental monograph *La Cellule Nerveuse* (in translation: *The Nervous Cell)* appears in Doin Publishing House in Paris (Figure 2), with an eloquent preface made by the famous Spanish father of Neuroscience, Santiago Ramón y Cajal, awarded with Nobel Prize in 1906. In the preface, Cajal speaks about Marinesco in the most eulogistic terms, due to his deep and sincere admiration for the work of the Romanian scientist. He considers Marinesco's work the most complete work on the neuron (“*résumé méthodique de l'état actuel de nos connaissances sur le neurone*,” p. VII) from all points of view (“*anatomique, physiologique et pathologique”*, p. VIII) recognizing not only Marinesco's unique capacities of biologist and of clinician but also his authority in a vast number of topics.

Marinesco's book is divided in two volumes: the first volume is dedicated to a general description of cellular structures with some connections to the neural peculiarities while the second and more important volume is dedicated entirely to the neuron. Marinesco's contributions are considered unique and valuable (“*la personnalité de l'observateur et du critique se manifeste tout entire”* … “*Il décrit admirablement les phénomènes”*, p. VIII). Among Marinesco's many important contributions from Cajal's point of view are the nervous ganglions grafts and mechanical compression effects on young animal ganglions (“*expériences qui prouvent la production expérimentale des expansions Nouvelles”*, p. X) qualifying Marinesco not only as a fine observer but also as a very creative experimentalist (“*l'auteur est tout à la fois un sagace observateur et un partisan convaincu et tenace de la méthode expérimentale”*, p. IX). Cajal recognizes Marinesco's steadfastness in supporting his theories with solid facts and observations (“à* force de sagacité et de persévérance parvient à imposer à la conviction générale la réalité d'un fait nouveau justement interprété”*, p. XIII) by which the Romanian neuroscientist contributes fundamentally to the neurology (“*l'édifice majestueux de la neurologie s'élève [grâce à] des livres tels que celui-ci”*, p. XIV) and opens new roads for the advancement of this field (“*signale en même temps les questions en litige et les lacunes recommandées à l'investigation future”*, p. XIV). According to the Canadian anatomist Murray: “La Cellule Nerveuse”, together with Cajal's better known “Histologie du Système Nerveux de l'Homme et des Vertébrés,” are the major chapters in the neurocytologist's Old Testament” (Barr, 1951).

## Marinesco's contributions are paradigm shifts in neuroscience

George Marinesco's contributions represent unique paradigm shifts in the field of Neuroscience. Here we mention just the salient ones. Marinesco was among the first medical doctors in the world who applied histochemical and electrophysiological methods in the field of neurology in scientific research. He brought original contributions to phenomena such as reflex trophicity, chromatolysis, neuronophagia, retrograde degeneration, as a consequence of the axon section. By ultra-microscopic research he applied colloid theory data to the structure of the neuron. Moreover, his daily contact with patients and his insight into the phenomenology of human brain made him use every one of the latest methods as they became available: the Roentgen ray, with which he investigated bone changes in acromegaly, the film camera, for the study of body movements in health and disease.

### Morphologist

Victor Babes was the first scientific personality in the formation of young Marinesco. That is where his knowledge and passion for microscopy comes from.

**Figure 3:**
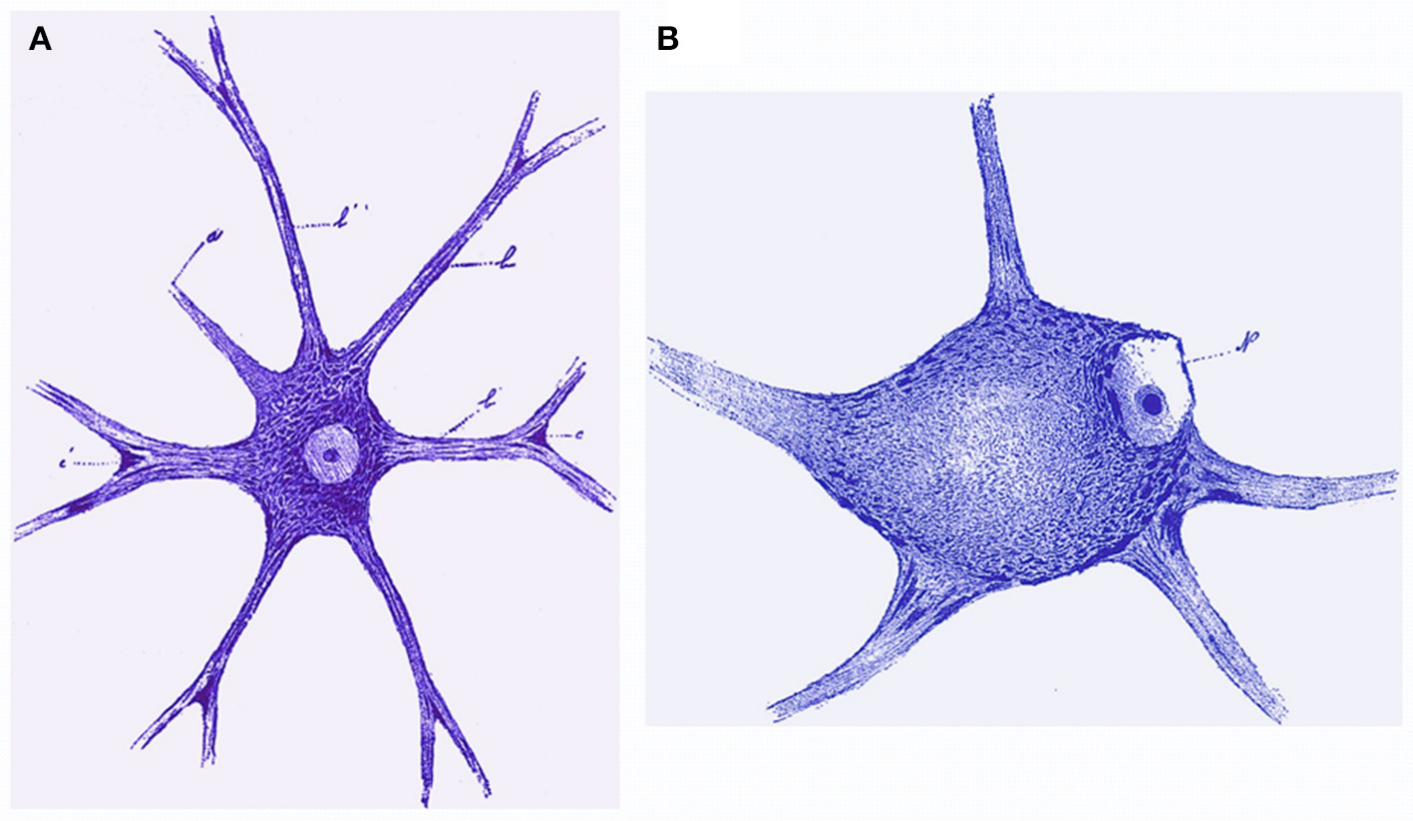
**(A)** Cell of the anterior horn in humans, Nissl staining. G. Marinesco, Polyneurtis characteristics at the level of nervous cells primary and secondary lesions (orig. Des polynévrites en rapport avec les lésions secondaires et les lésions primitives des cellules nerveuses), Rev Neurol, 1896, 4e année, n° 5 (15 mars), Figure 13. **(B)** Central chromatolysis. Cell of the anterior horn in a case of polyneuritis. Lesions are identical to those observed after a nerve section. G. Marinesco, Polyneurtis characteristics at the level of nervous cells primary and secondary lesions (orig. Des polynévrites en rapport avec les lésions secondaires et les lésions primitives des cellules nerveuses), Rev Neurol, 1896, 4e année, no. 5 (15 mars), Figure 17.

**Figure 4:**
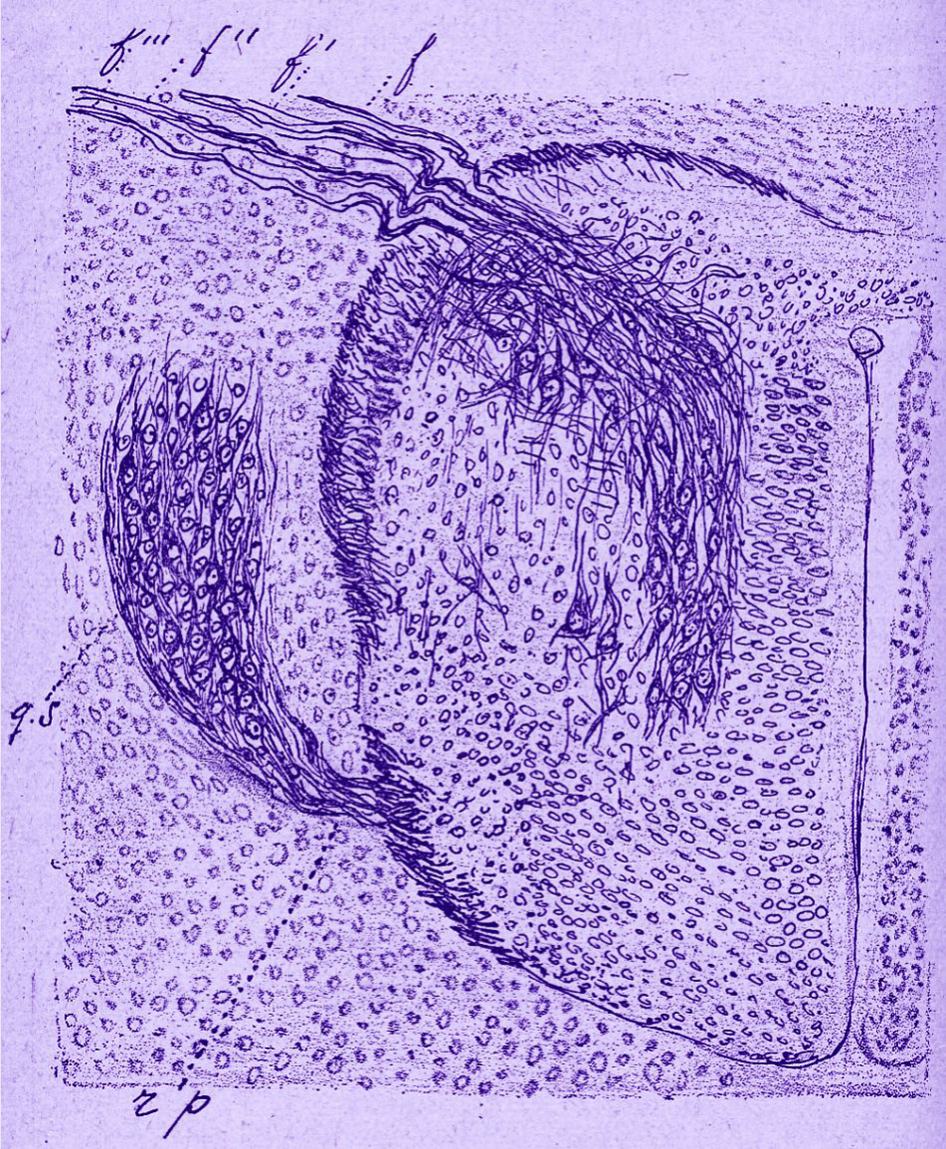
Reproduction of Figure 76 (p. 356) from the first volume of “La Cellule Nerveuse” (Marinesco, 1909, p. 29). This figure represents a transversal section of a histological preparation showing the nervous fibrils. This is from a chick embryo at 6 days of incubation. Ventral region is upside. “f” is for ventral roots, “gs” for spinal ganglion, and “rp” for dorsal roots.

**Figure 5:**
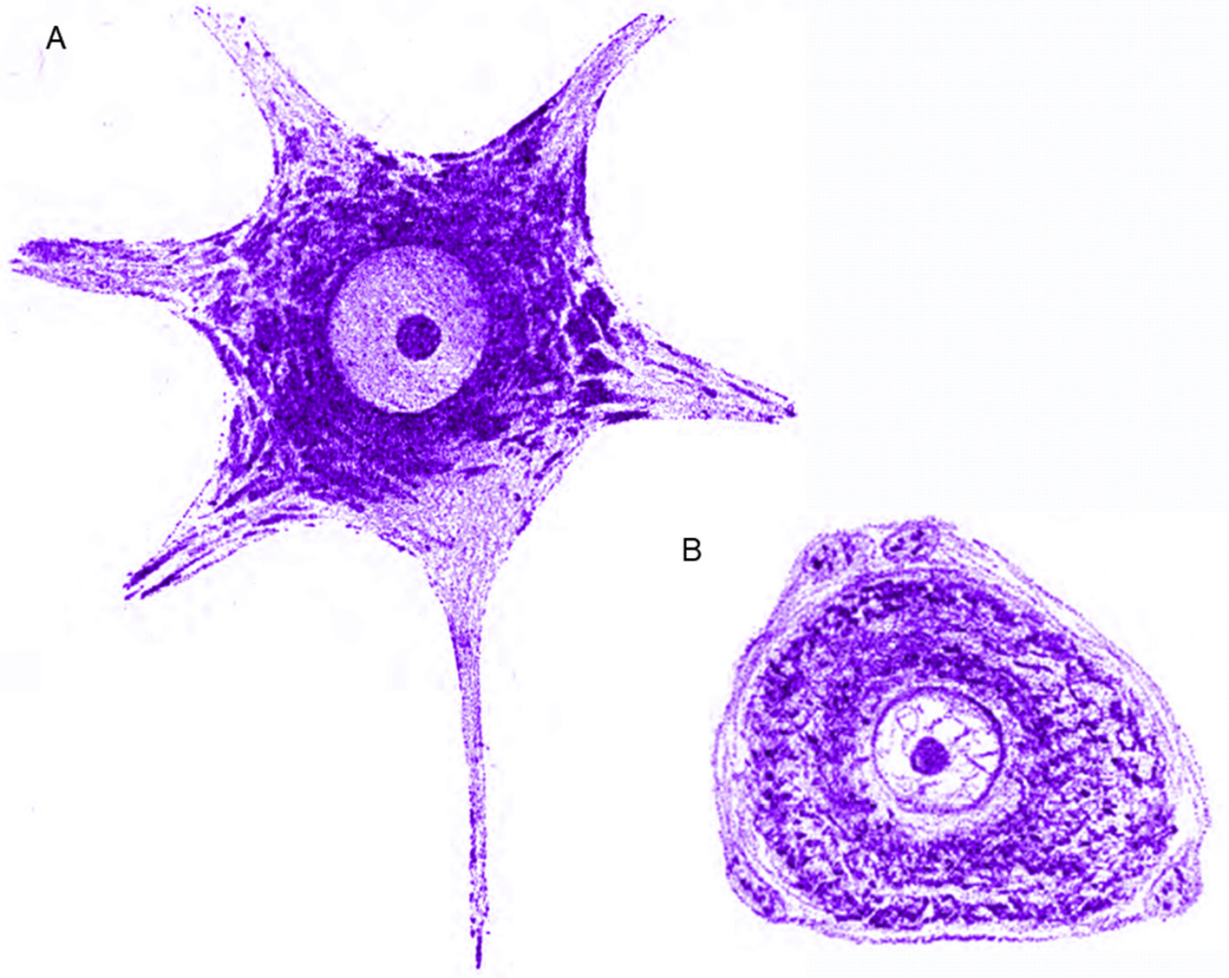
**(A)** Normal root cell of the lumbar spine of a rabbit. The chromatophilic substance is disposed in the corpuscular form of the gonals having an outer concentric orientation of nucleus. In the protoplasmic extensions they are in the form of sticks having the same direction as the path of the extensions (Adjusted from Marinesco's book “La Cellule Nerveuse” Figure 17). **(B)** The clear cell of the average volume of the chromatophilic substance is disposed in two layers, one peripheral and the other peri-nuclear. They are separated by a lighter intermediate zone due to the poor chromatophilic substance in this zone. (Adjusted from Marinesco's book “La Cellule Nerveuse” Figure 16).

**Figure 6:**
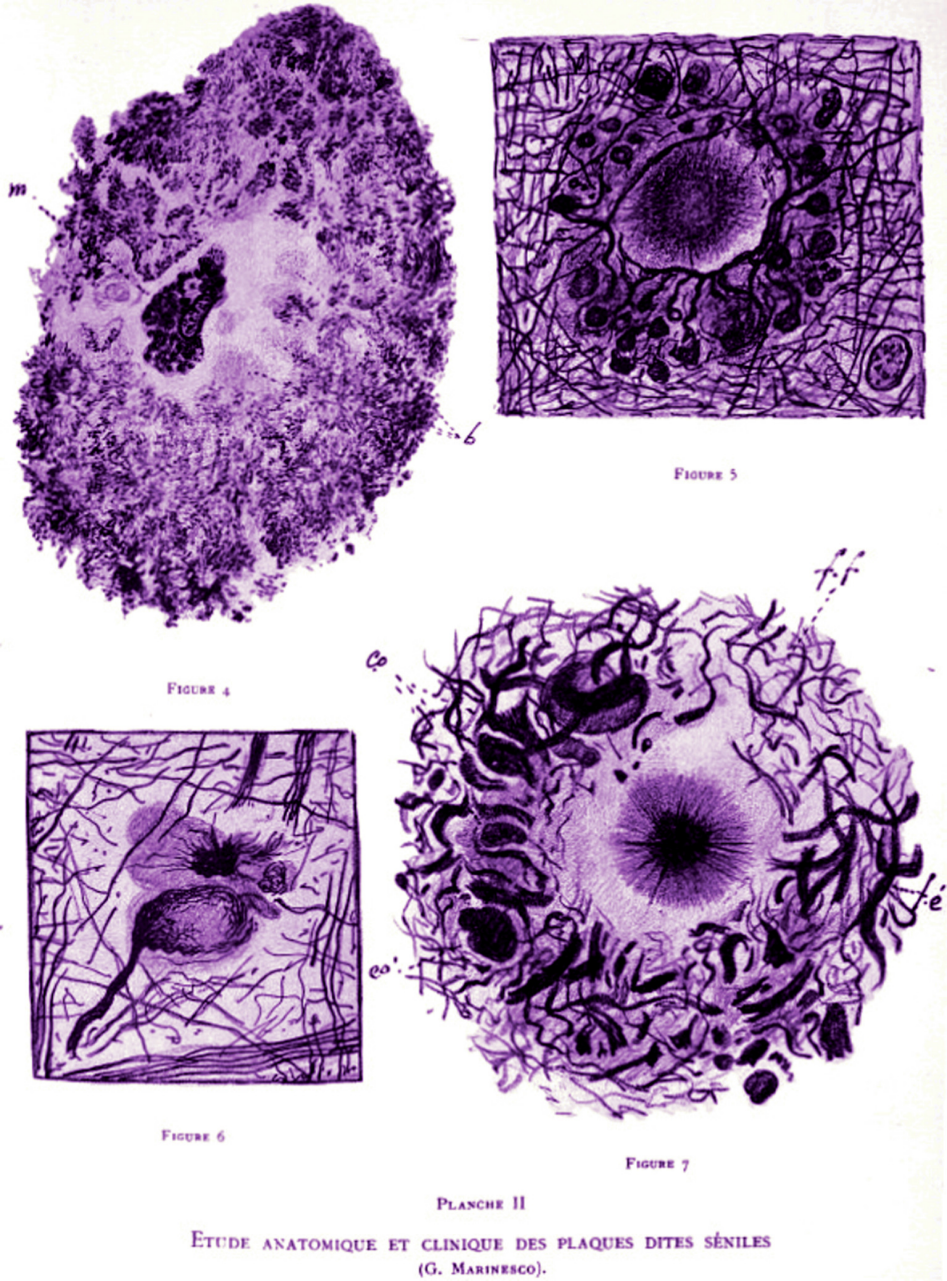
Anatomical and clinical study of the senile plaques, Marinesco (1912), Encéphale (Paris), 1er semestre, p. 105–132. Reproduction of the plate II. Figure 4 Plaque with a large zonal layer constituted by an aggregation of filamentous material. A macrophage (m) lies in the center of the plaque and near the colorless and amorphous region. Figure 5 shows a plaque with a bulky central nucleus and typical phenomena of neurotisation. The fibers coming from the new formation penetrate into the plaque. They arrive at the vicinity of the nucleus, they surround it and give off collaterals that end each with a button and form a rosette. Figure 6 Fiber with a terminal ball. The black precipitate could be evidenced near it. The ball is degenerating. Figure 7 Plaque with a central nucleus constituted by an argyrophilic central zone and a radial peripheral zone. The zonal layer is made up by fibers that are short, thick, sometimes thin at their extremity, undulated; by other thinner fibers (f, f) that creep into the first ones (f, e) forming ribbons and argentophilic corpuscles (ca, ca') around the central nucleus.

For a modern reader, the most striking characteristics are the illustrations with drawings resembling the modern computer enhancement or hyperrealism (Figures 3–6). Morphology of nervous cells (axon growth cones, Ramón y Cajal, 1890a,b) is further detailed at subcellular level that includes the fine structure corresponding to Golgi organ, neurotubules, mitochondria, and other structures, etc. However, the functional link between neurons (Figure 3) in Marinesco's view supports the notion of contiguity (neuron doctrine) and not that of continuity (reticular theory), in favor of Cajal and contrary to Golgi. Limited only to optic microscopy observation of classical stain methods, he is not only describing but he is trying to find function supported by structure. Neurofibrils (Figure 4) are “*axonal neurotubuls involved in conduction of nerve impulses*” (Marinesco, 1914). He is concerned with “chromatophilic elements” (Figure 5) grounding different hypotheses. He is focusing on the propagation of nervous impulse, use of the Cajal notion of “dynamic polarization” and elaborate it (Marinesco, 1909).

Direct observation of living structure, by Lepine and Duval (Ramon y Cajal, 1909), through optic microscope opened a new horizon; they extrapolated white cells amoeboidal travel through tissues to nervous system building the theory of amiboism (or amoebic movement) and neural plasticity. They explained sleep and sudden stop of activity such as hysterical palsy by this phenomenon (Marinesco and Nicolesco, 1933).

Chemotactic growth and orientation of neuronal structures make possible the genesis of nervous system and its regeneration after Marinesco (in concordance with Ramón y Cajal, 1893; de Castro et al., 2007). Also, Marinesco notice that not all neurons are appearing simultaneously but progressively till a certain stage of development depending on species. There are multiple contributions of Marinesco from studies on frozen nerves and multiple sclerosis to many other areas of investigation, mentioned in the paradigm shifts paragraph (Marinesco and Draganesco, 1929; Marinesco et al., 1931a,b; Marinesco, 1933, 1936, 1937, 1963a,b; Marinesco and Goldstein, 1933; Potemkina, 1963; Petresco, 1964; Shpilmann, 1964; Popescu et al., 2003; Catala and Poirier, 2012).

After almost eight decades since he passed away, it is still difficult to make a hierarchy of his contribution to science. The order of his achievements is not pretending to be grounded objectively, but could be the consequence of author subjective perception and impressions, and last but not least, on memories transmitted by coworkers, pupils and friends orally. That is the reason why some statements are not cited, but introduced as text.

### Landmark discoveries of marinesco

#### Locus niger (substantia nigra) as a possible cause in parkinson disease

Marinesco published (early in his career) an extremely necessary atlas (Marie, 1896; Marinesco, 1896a,b) on the pathological histology of the nervous system, together with the Romanian bacteriologist Victor Babes and the French pathologist Paul Oscar Blocq (Blocq and Marinesco, 1892; Hostiuc et al., 2016). His description with Blocq, of a case of Parkinsonian tremor due to the substantia nigra tumors in 1893, was the basis for Édouard Brissaud's theory that Parkinsonism occurs as a consequence of damage to the substantia nigra (Blocq and Marinesco, 1893). Marinesco's team is demonstrating for the first time the role of locus niger/substantia nigra in the pathogenesis of parkinsonism (Percheron et al., 1994; Quan et al., 2001a,b; Parent and Parent, 2010).

#### Marinesco bodies

Marinesco bodies are nuclear inclusions found in pigmented neurons of the substantia nigra and locus ceruleus of humans and monkeys (Leestma and Andrews, 1969; Hirai et al., 1977; Resl, 1982; Siddiqi and Peters, 1999; Fujigasaki et al., 2001; Beach et al., 2004; Grigoriev et al., 2015; Grigoriyev et al., 2015). Structures such as locus coeruleus and substanta nigra contain inclusions named by him paranucleolar corpuscles, become a subject of debate (Yuen and Baxter, 1963; Janota, 1979; Kettner et al., 2002; Kumada et al., 2002; Woulfe et al., 2004; Kanaan et al., 2007; Krygowska-Wajs et al., 2008; Jyothi et al., 2015; Abbott et al., 2017). They are known as Marinesco bodies (Okamoto and Hirai, 1981; Ono et al., 1989; Odagiri et al., 2012) presenting a pathological significance as Lewy bodies (Beach et al., 2004; Kon et al., 2014).

#### Senil plaques

Senile plaques (Figure 6) are “extracellular deposits of beta amyloid” in the gray matter of the brain (Marinesco, 1912, 1928). In 1892 George Marinesco and Paul Blocq have described for the first time the presence of senil plaque deposits in the gray matter (Blocq and Marinesco, 1892; Buda et al., 2009). In 1906 Alois Alzheimer discovered the connection between senile plaques and dementia (Alzheimer, 1907). It sounds like the infectious roots of Alzheimer's are buried deep into the past (Marinesco, 1912; Broxmeyer, 2017).

#### Marinesco-radovici sign

Marinesco-Radovici sign or the palmomental reflex (PMR) is a “primitive reflex manifested by the ipsilateral mentalis muscle twitching when applying moderate pressure on the palmar ternar eminence” (Marti-Vilalta and Graus, 1984). It is an example of a “frontal release sign” (Marinesco and Radovici, 1920). Usually present in the infancy it gradually disappears during childhood to reappear later in life in elderly. Its reappearance is the sign of cortical inhibitory pathway disruptions located especially in the frontal lobe (Ladino et al., 2015) encountered in congenital conditions such as Down's, in neurodegenerative diseases such as Alzheimer's or in stroke (Owen and Mulley, 2002).

#### Marinesco-sjögren syndrome

MSS is a rare congenital disorder with the most common symptom being the “*difficulty to coordinate voluntary movements due to muscular atrophy*,” hypotonia, “cerebellar ataxia” and “*cataracts associated to delayed psychomotor development*,” short stature and frequent “hypergonadotropic hypogonadism” with overall non-affected lifespan (Mahloudji et al., 1972; Skree, 1981; Bromberg et al., 1990; Katafuchi et al., 1990; Anttonen and Lehesjoki, 1993; Brogdon et al., 1996; Sakai et al., 2008).

#### Confirmed the discovery of treponema pallidum

Confirmed the Discovery of *Treponema pallidum* in the brain of patients with general paresis (Marinesco and Minea, 1913). Marinesco described the neurological manifestations such as tabes dorsalis consisting of spinal cord wasting in advanced syphilis linking them to the observation of the degeneration of the central processes of dorsal root ganglions confirming in this way early discoveries by Hideyo Nogouchi (Noguchi and Moore, 1913; Miklossy, 2008).

#### The use of cinematography in the first science movies

During years 1898 to 1901, Marinesco made “the first science movies in the world” in his clinic in Bucharest (Cantacuzene, 1973). *The walking troubles of organic hemiplegy* (1898), *The walking troubles of organic paraplegies* (1899), *A case of hysteric hemiplegy healed through hypnosis* (1899), *The walking troubles of progressive locomotion ataxy* (1900), and *Illnesses of the muscles* (1901). Professor Marinesco named his works “studies with the help of the cinematograph”, and published the results, along with several consecutive frames, in issues of *La Semaine Médicale* magazine from Paris during 1899–1902.

In 1924, Auguste Lumière recognized the priority of professor Marinesco concerning the first science films: “I've seen your scientific reports about the usage of cinematograph in studies of nervous illnesses, when I was still receiving La Semaine Médicale, but back then I had other concerns, which left me no spare time to begin biological studies. I must say I forgot those works and I am thankful to you that you reminded them to me. Unfortunately, not many scientists have followed your way.”

### Relevant work

He carries out research on a wide variety of topics, the results of which appear in numerous papers such as *Histochemical Research on Oxidative Fertilizers in the Life Stories* (1924), Old Age and Rejuvenation (1929), Conditional Reflexes (1935, together with Arthur Kreindler). Le Tonus des Muscles striés (1937, together with Nicolae Ionescu-Siseşti, Oskar Sager and Arthur Kreindler, prefigured by the famous neurophysiologist Sir Charles Sherrington), Determinism and Causality in Biology (1938). Besides monographs, he has published over 1,000 articles in specialized journals. To this activity is added the participation in numerous congresses and scientific meetings, to which he was often the main reporter.

## National and international recognition

On the assignment of Pierre Marie, he lectured on the pathological anatomy of acromegaly at the Berlin International Congress in 1890.

In 1897 he defends at the Faculty of Medicine in Paris the Ph.D. dissertation entitled Juicy Hand in Syringomielia. In the same year—returned to the country—received the post of Head of the Nervous Disease Service at Pantelimon Hospital; A year later he is appointed professor at the Nervous Disease Clinic of the Faculty of Medicine in Bucharest.

He is becoming increasingly known and appreciated in the international scientific circles; In 1912 he was elected a correspondent member of the Paris/French Academy of Medicine.

In 1925 on the 100th anniversary of Charcot's birth, Marinesco was chosen from all the disciples to evoke the personality of the great master.

- *Member in Romanian Academy*. In 1906 he is elected as full member of the Romanian Academy, where he presented his acceptance speech to the Romanian Academy entitled *Progresses and Trends of Modern Medicine* (Hostiuc et al., 2016).
- *Marinesco was elected in the Paris Academy of Medicine*. In 1912 Marinesco was elected as corresponding member of the Academy of Medicine where his eulogy is pronounced by Louis Ribadeau Dumas (1938) (Ribadeau Dumas, 1938).
- *Marinesco was a member of the Royal College of Physicians of London* (Catala and Poirier, 2012).
- He was decorated in 1930 with the “Workmanship Medal” Class 1 for education, for the tasks of higher education for 10 years and in 1936 was decorated with the rank of Commander of the Romanian Crown.
- *Marinesco was very appreciated by Santiago Ramón y Cajal, who proposed and recommended him for the award of the “Mart*í*nez y Molina prize”, granted by the Royal Academy of Medicine of Spain* (Courtesy of anonymous reviewer that read the letters of Cajal, preserved at the Cajal Institute, in Madrid).
- *Marinesco was received in Argentina as the Prince of Neurology*. It is important to remember his visit in Argentina, in Buenos Aires, where he received protocols almost as a state president. Population crowded on streets yelling: “*Wellcome, Prince of Neurology*.” Many families brought patients in severe advanced states to him with hope of healing. When he did not succeed, he did what he already had done home: good words and encouragement.

## Conclusion

George Marinesco is the founder of Romanian School of Neurology and one of the most remarkable neuroscientists of the last century. Marinesco was a broadly minded neuroscientist with an integrative view and a wide range of research interests, including pathological anatomy, neurophysiology, and experimental neuropathology (Marinesco, 1895, 1902; Marinesco et al., 1937; Chudley, 2003). His work had a single limitation due to language by not having an English translation available published till now. Nevertheless, his brilliance remains in the constellation if modern neuroscience.

## Author contributions

All authors listed have made a substantial, direct and intellectual contribution to the work, and approved it for publication.

### Conflict of interest statement

The authors declare that the research was conducted in the absence of any commercial or financial relationships that could be construed as a potential conflict of interest.
